# The mental health of university students during the COVID-19 pandemic: An online survey in the UK

**DOI:** 10.1371/journal.pone.0262562

**Published:** 2022-01-12

**Authors:** Tianhua Chen, Mike Lucock

**Affiliations:** 1 Department of Computer Science, School of Computing and Engineering, University of Huddersfield, Huddersfield, West Yorkshire, United Kingdom; 2 Centre for Applied Research in Health, School of Human and Health Sciences, University of Huddersfield, Huddersfield, West Yorkshire, United Kingdom; Satyawati College (Eve.), University of Delhi, INDIA

## Abstract

Higher education students’ mental health has been a growing concern in recent years even before the COVID-19 pandemic. The stresses and restrictions associated with the pandemic have put university students at greater risk of developing mental health issues, which may significantly impair their academic success, social interactions and their future career and personal opportunities. This paper aimed to understand the mental health status of University students at an early stage in the pandemic and to investigate factors associated with higher levels of distress. An online survey including demographics, lifestyle/living situations, brief mental well-being history, questions relating to COVID-19 and standardised measures of depression, anxiety, resilience and quality of life was completed by 1173 students at one University in the North of England. We found high levels of anxiety and depression, with more than 50% experiencing levels above the clinical cut offs, and females scoring significantly higher than males. The survey also suggested relatively low levels of resilience which we attribute to restrictions and isolation which reduced the opportunities to engage in helpful coping strategies and activities rather than enduring personality characteristics. Higher levels of distress were associated with lower levels of exercising, higher levels of tobacco use, and a number of life events associated with the pandemic and lockdown, such as cancelled events, worsening in personal relationships and financial concerns. We discuss the importance of longer-term monitoring and mental health support for university students.

## Introduction

The mental well-being of higher education students was a growing concern even before the COVID-19 pandemic, with increasing numbers of students experiencing mental health problems as reported by UK Parliament Briefing Paper [1]. Community surveys suggest that common mental health problems, anxiety and depression, generally affect one in six people in a given week in England [2], and concerns were expressed early in the pandemic about the mental health impact of the pandemic on the general population [3], at least in the short term. For higher education students, the pandemic presented a number of specific challenges, such as the transfer of more learning and support services online, which many students found difficult to engage effectively [4], leading to increased anxiety and concerns about their academic performances and long-term employment [5, 6]. Other impacts include the closure of student halls, cancellation of exchange studies and graduation ceremonies, loss of part-time jobs, and increased uncertainties regarding career options. The lockdown and social distancing measures also led to limited opportunities for socialising and establishing relationships, with greater reliance on social media, and possible chronic loneliness brought by social isolation [7].

A number of studies have explored the impact of the pandemic on the mental health of university students and factors associated with higher levels of distress. For example, a US interview survey of 195 undergraduate students from one university [8] reported negative impacts of the COVID-19 pandemic and the urgent need to develop interventions and preventive strategies. Another US survey of 162 undergraduates [9] found high levels of mental health distress, with depression being associated with difficulties focusing on academic work and loss of employment and higher levels of anxiety more likely in students who spent more than an hour per day looking for information on COVID-19. An online survey of 255 students at a university in Hong Kong in July 2020 also found high levels of depression with perceived available peer support being negatively associated with depressive symptoms [10]. Another cross-sectional web-based study of 324 college students in India between November and December 2020 [11] suggested that 68.8% had high fear of COVID-19, 28.7% had moderate to severe depression, and 51.5% had mild to severe anxiety, with having a family member who was infected with COVID-19 being significantly associated with anxiety and depression. Studies have also provided evidence of a worsening of common mental health problems and wellbeing during the pandemic. For example, a survey of undergraduate students by the Higher Education Policy Institute in the UK found that 58% reported a worsening in their mental health because of the pandemic, 14% said it was better and the remaining 28% said it was the same [12]. Also in the UK, a survey of students in higher and further education conducted by the National Union of Students, found that 52% described their current mental health and well-being as worse, 35% described it as the same and 8% as better, compared with their life before the pandemic [13]. The Student Covid Insight Survey (SCIS), conducted in November 2020, found 57% of students reported that their well-being and mental health had become slightly or much worse since the start of the autumn term [14], with lower levels of life satisfaction and happiness, and higher levels of anxiety, compared with the general population.

Longitudinal studies comparing mental health before and during the pandemic are rare but a study of 254 undergraduates at one UK university [15], found a significant increase in depression and reduction in wellbeing during the first lockdown (April/May 2020) compared with before the pandemic (autumn 2019) and that over a third of the sample could be classed as clinically depressed at lockdown, an increase from 15% before the pandemic. The increase in depression was highly correlated with a worse sleep quality. A longitudinal survey of 66 students in a Chinese college also concluded that sleep quality was a key factor in the emotional impact on students, and that daily physical activity and good sleep may mitigate mental health problems [16]. Interestingly, they also found reductions in people’s aggressiveness, which they suggested was due to people realizing the fragility and preciousness of life.

These studies have contributed to our understanding of the impact of the pandemic and lock-down on students’ mental health, but they have tended to involve relatively small sample sizes of undergraduates and have not looked at positive factors associated with better mental health, such as resilience. This paper describes a relatively large survey of undergraduate and postgraduate students which investigates different aspects of mental health and coping, including anxiety, depression, the resilience to cope with difficulties, quality of life and general health, and a range of questions on demographics, lifestyle/ living situation and COVID-19 related factors. Conducted at a university in the UK, this study aimed to identify: 1) The impact of the COVID-19 pandemic on University students; 2) Levels of mental health and quality of life in University students during the COVID-19 pandemic; 3) Predictors of mental health and quality of life.

## Materials and methods

### Design and setting

This was a cross-sectional on-line survey at a large university in the North of England, UK. Almost 20,000 students attend the University each year. The study was approved by a University ethics committee. The data collection was conducted in the period between 26.06.2020 and 30.07.2020. To put this in context with the lockdown in England, it entered the first national lockdown on 23.03.20 and lockdown measures were eased on 01.06.20.

### Participants

Students, including both undergraduates and postgraduates across all seven schools at the university were eligible and sent an invitation by local school administrators through their mailing-list.

### Procedure

A link to the survey that was deployed in Google Forms was delivered to students via e-mail. The anonymous survey questionnaire took up to 10 minutes to complete, once participants agree to take part. Students completing the survey were entered into a prize draw for £650 worth of gift vouchers, distributed in varying amounts to 36 prize winners.

### Measures

The following set of demographic information and measures are used to identify the students’ mental health, wellbeing and resilience.

#### Demographics

The first part of survey included demographic information including age, gender, ethnicity, current educational level, and relationship status.

#### Patient health questionnaire (PHQ-9) [17]

The PHQ-9 is a self-administered screening questionnaire, validated for use in primary care and community settings, to measure the severity of depression. Nine questions cover different aspects of depression on a four-point scale—“0” (not at all), “1” (several days), “2” (more than half the days), “3” (nearly every day). Scores can be categorised as 0-4 none, 5-9 mild, 10-14 moderate, 15-19 moderately severe, 20-27 severe. The total score was used as the dependent variable with the aim to conduct statistical significance test for alternative independent variables.

#### Generalised anxiety disorder questionnaire (GAD-7) [18]

The GAD-7 is also a self-administered screening questionnaire, which measures the severity of generalised anxiety disorder (GAD). Seven questions are rated on the same four-point scale as the PHQ-9. Scores of 5, 10, and 15 are taken as the cut-off points for mild, moderate and severe anxiety, respectively. In this study the total score was be used as the dependent variable to conduct statistical significance test.

#### Lifestyle / Living situation under COVID-19

This set of questions explored the lifestyle, living situation, behaviours and experiences of individuals. These included experiences of adversities (e.g., worsen personal relations/financial situation/live conditions, loss of employment/families etc.), and the frequency of conducting exercise/ communicating with friends/relatives and the 122 consumption of alcohol/tobaccos during the lockdown period. The values of these variables were dichotomised to 0 (Never, Rarely, Sometimes) and 1 (Often, Always) for 124 analysis [9].

#### Brief resilience scale (BRS) [19]

The BRS aims to measure the ability to bounce back or recover from stress. It has a 5-point Likert response scale, for six items, ranging from 1 = strongly disagree to 5 = strongly agree, with three items positively phrased and three negatively phrased. The BRS is scored by reverse coding items 2, 4, and 6, and then calculating the mean of the six items. An averaged score of 1.00 to 2.99 suggests low resilience, 3.00 to 4.30 normal resilience and 4.31 to 5.00 high resilience. Similar to PHQ-9 and GAD-7, the overall averaged score was used as the dependent variable to conduct statistical significance test.

#### Brief mental wellbeing history

Three questions asked about the students’ history of treatment and support for a mental health issue, including therapy and medication.

#### EQ-5D-5L

The EQ-5D-5L instrument [20] is a self-assessed, health related, quality of life questionnaire. The descriptive system comprises five dimensions: mobility, self-care, usual activities, pain/discomfort and anxiety/depression. Each dimension has 5 levels: no problems, slight problems, moderate problems, severe problems and extreme problems. Responses are coded as single-digit numbers expressing the severity level selected in each dimension. The overall score will be used as a dependent variable, with the best health state (11111) being a score of 5 and the worst health state (55555) being a score of 25. Additionally, the EQ-VAS was also used for students to provide a broad self assessment of their health, on a visual analogue scale ranging between 100 (best imaginable health) and 0 (worst imaginable health).

#### COVID-19 related questions

A set of five questions were asked in relation to COVID-19 pandemic. These included: how often the person practised the recommended social distancing on a 5-point scale (‘1’ is never and ‘5’ is always); the severity of the risk group the subject assumes they belong to; whether the subject is cohabiting with anyone falling with the risk groups; how likely the subject feels at the risk of contracting COVID-19 (on a 5-point scale where ‘1’ is definitely not and ‘5’ is certain); the extent to which the subject had felt needing extra support during lockdown (where ‘0’ was no need for extra support and ‘100’ indicated immediate support required).

### Data analysis

All analysis below used the SciPy (Scientific Python) [21], which is a free and open-source Python library extensively used for scientific and technical computations for data science. Descriptive analyses examined the distribution of all variables/questions of interest, with the summarised information presented in Tables 1–5. A bivariate analysis was then conducted to examine the associations between each of the independent variables against each of the six decision variables that had been previously introduced, including PHQ9 depression, GAD 7 Anxiety, BRS6 Resilience, EQ-5D-5L life quality, self-rated health score and support needs. Specifically, paired two tail t-tests were used to examine the significance of the associations. The findings are presented in Tables 6–9, where the significance levels are represented as *ns*(*p* > 0.05), *(*p* ≤ 0.05);**(*p* ≤ 0.01);***(*p* ≤ 0.001) on the basis of the associated *p* values. Furthermore, a multivariate linear regression was utilised to evaluate the predictive capabilities of the independent variables that have achieved statistical significance (i.e., *p* < 0.05) with respect to the corresponding decision variable.

**Table 1:** Demographics descriptive analysis.

Question/Variable	Choice	N (%)
1 Age	mean ± std	25.7 ± 8.9
	median	22
2 Gender	Female	826 (70.4%)
	Male	340 (29.0%)
	Others	7 (0.6%)
3 Residence	Huddersfield	397 (33.8%)
	Bradford	80 (6.8%)
	Leeds	79 (6.7%)
	Manchester	64 (5.5%)
	Wakefield	51 (4.4%)
	Others	502 (42.8%)
4 Ethnic origin	White	788 (67.2%)
	Non-white	385 (32.8%)
5, Education level	Undergrads	551(47.0%)
	Postgrads	622(53.0%)
6, Relationship	Single	529 (45.1%)
	Non-single	63 5(54.1%)
	Others	9 (0.8%)

**Table 2:** Lifestyle / Living situation descriptive analysis.

Question/Variable	Choice	N (%)
1 During the COVID-19 pandemic, have you suffered from any of the following situations?	Worse personal relations	471 (40.2%)
	You or a loved one requiring hospitalisation	159 (13.6%)
	Worse living conditions	145 (12.4%)
	Loss of employment by you or your partner	201(17.1%)
	Worse financial situation	464(39.6%)
	Cancellation of an important event	658(56.1%)
	Death of a partner / close relative / friend	157(13.4%)
	None of the above	194(13.4%)
2 How often have you been exercising during the lockdown period?	Very Often/Often	335(30.3%)
3 How often have you consumed alcohol during the lockdown period?	Always/Often	211(18.0%)
4 How often have you smoked tobacco during the lockdown period?	Always/Often	141(12.0%)
5 Has the current COVID-19 outbreak impacted your relationships with your friends or family?	Yes/Maybe	848 (72.3%)
6 How often have you been communicating with friends / family through e.g., voice/video calls, social media during the lockdown period?	Always/Often	709(60.4%)

**Table 3:** Brief mental wellbeing history descriptive analysis.

Question/Variable	Choice	N (%)
1 Have you ever been referred to, or participated in talking therapies for a mental health issue including cognitive behaviour therapy, counselling etc.?	Yes	475 (40.5%)
2 Have you ever, or are you currently, taking medications for a mental health issue?	Yes, Previously/ Currently	325(27.7%)
3 Have you accessed or attempted to access healthcare services for yourself or a member of your family during the pandemic?	Yes	401(34.2%)

**Table 4:** Covid related questions for descriptive analysis.

Question/Variable	Choice	N (%)
1 During the COVID-19 pandemic how often have you practised the recommended social distancing guidelines provided by the Government?	Always/Often	1096 (93.4%)
2 Which of these 3 COVID-19 risk groups do you do you believe you are under?	High/increased risk	175(14.9%)
3 Are you cohabiting with another person that falls within any of these risk groups?	Yes	353(30.1%)
4 How likely do you feel you are at risk of contracting COVID-19	Likely/Extremely likely	194(16.5%)

**Table 5:** Descriptive analysis on decision variables (N = 1173).

Variable	Mean ± STD
PHQ9 Depression	10.91 ± 6.18
GAD7 Anxiety	8.87 ± 5.80
BRS6 Resilience	3.06 ± 0.31
EQ5D5L Quality of Life	7.91 ± 2.76
Health score	69.51 ± 20.62
Support urgency	29.92 ± 30.08

**Table 6:** Bivariate analysis on demographics.

Variables	Categories	PHQ9 Depression	GAD7 Anxiety	BRS6 Resilisence	EQ5D5L Life quality	Health score	Support urgency
I. Demographics							
Gender (N = 1166)	Male = 340 (29.2%)	9.74 ± 6.46	7.69 ± 5.82	3.06 ± 0.33	7.68 ± 2.92	71.84±19.40	30.38 ±29.46
	Female = 826 (70.8%)	11.36 ± 5.97	9.35 ± 5.71	3.05 ± 0.30	7.98 ± 2.70	68.52±21.08	28.41 ±31.38
	T-test significance	***	***	ns	ns	**	ns
Ethnicity	White = 788 (67.2%)	10.74 ± 6.08	8.85 ± 5.76	3.04 ± 0.29	7.94 ± 2.70	70.26 ± 19.66	28.48 ± 29.76
	Non-White = 385 (32.8%)	11.24 ± 6.36	8.91 ± 5.88	3.09 ± 0.35	7.84 ± 2.85	67.97 ± 22.40	32.85 ± 30.56
	T-test significance	ns	ns	**	ns	ns	*
Education	Undergrad = 551 (47.0%)	11.44 ± 6.41	9.13 ± 5.89	3.06 ± 0.28	7.88 ± 2.65	69.30 ± 19.97	29.01 ± 29.38
	Postgrad = 622 (53.0%)	10.44 ± 5.93	8.64 ± 5.71	3.05 ± 0.34	7.94 ± 2.85	69.70 ± 21.20	30.72 ± 30.68
	T-test significance	**	ns	ns	ns	ns	ns
Relationship (N = 1164)	Single = 529 (45.4%)	11.17 ± 6.29	8.79 ± 5.75	3.07 ± 0.34	7.86 ± 2.77	68.59 ± 21.29	28.83 ± 29.64
	Non-Single = 635 (54.6%)	10.71 ± 6.05	8.95 ± 5.84	3.05 ± 0.29	7.95 ± 2.73	70.45 ± 19.83	30.77 ± 30.41
	T-test significance	ns	ns	ns	ns	ns	ns

**Table 7:** Bivariate analysis on lifestyle / living situations.

Variables	Categories	PHQ9 Depression	GAD7 Anxiety	BRS6 Resilience	EQ5D5L Life quality	Health score	Support urgency
II. Lifestyle / living situation							
Adversity	Some suffering = 979 (83.5%)	10.80 ± 6.14	8.76 ± 5.82	3.06 ± 0.32	7.91 ± 2.80	70.26 ± 20.01	29.01 ± 29.74
	None = 194 (16.5%)	11.45 ± 6.36	9.40 ± 5.66	3.01 ± 0.25	7.90 ± 2.54	65.75 ± 23.16	34.48 ± 31.44
	T-test significance	ns	ns	*	ns	**	*
Exercise	Never/Rarely/Sometimes = 838 (69.7%)	11.76 ± 6.19	9.38 ± 5,77	3.06 ± 0.31	8.21 ± 2.87	66.84 ± 20.98	32.02 ± 30.59
	Very often/Always = 335 (30.3%)	8.76 ± 5.59	7.59 ± 5.68	3.04 ± 0.31	7.14 ± 2.28	76.18 ± 18.07	24.66 ± 28.14
	T-test significance	***	***	ns	***	***	***
Alcohol	Never/Rarely/Sometimes = 962 (82.0%)	10.95 ± 6.20	8.87± 5.79	3.06 ± 0.31	7.95 ± 2.81	69.47 ± 20.76	29.69 ± 30.04
	Very often/Always = 211 (18.0%)	10.72 ± 6.08	8.83 ± 5.86	3.04 ± 0.29	7.73 ± 2.49	69.67 ± 20.04	30.96 ± 30.31
	T-test significance	ns	ns	ns	ns	ns	ns
Tobacco	Never/Rarely/Sometimes = 1032 (88.0%)	10.57 ± 6.04	8.53± 5.70	3.05 ± 0.31	7.77 ± 2.67	70.49 ± 20.07	28.92 ± 29.65
	Very often/Always = 141 (12.0%)	13.38 ± 6.58	11.33 ± 5.95	3.07 ± 0.29	8.92 ± 3.09	62.28 ± 23.08	37.21 ± 32.28
	T-test significance	***	***	ns	***	***	**
Relationship impact	Yes/Maybe = 848 (72.3%)	12.14 ± 6.05	10.04± 5.67	3.06 ± 0.32	8.38 ± 2.84	66.64 ± 20.89	33.91 ± 30.32
	No = 325 (27.7%)	7.70 ± 5.29	5.80 ± 4.96	3.05 ± 0.31	6.68 ± 2.08	76.98 ± 17.89	19.49 ± 26.3
	T-test significance	***	***	ns	***	***	***
Communication	Never/Rarely/Sometimes = 464 (39.6%)	12.63 ± 6.36	10.49± 5.82	3.04 ± 0.32	8.56 ± 3.11	64.77 ± 21.88	35.51 ± 32.59
	Very often/Always = 709 (60.4%)	9.78 ± 5.79	7.80 ± 5.54	3.07 ± 0.31	7.48 ± 2.41	72.62 ± 19.14	26.26 ± 27.74
	T-test significance	***	***	ns	***	***	***

**Table 8:** Bivariate analysis on brief mental wellbeing history.

Variables	Categories	PHQ9 Depression	GAD7 Anxiety	BRS6 Resilisence	EQ5D5L Life quality	Health score	Support urgency
III. Mental wellbeing history							
Talking therapy	Yes = 475 (40.5%)	12.80 ± 6.22	10.88 ± 5.70	3.07 ± 0.28	9.06 ± 3.08	63.35 ± 22.61	37.20 ±31.32
	No = 698 (59.5%)	9.62 ± 5.81	7.50 ± 5.47	3.05 ± 0.33	7.12 ± 2.20	73.70 ± 18.00	24.96 ±28.18
	T-test significance	***	***	ns	***	***	***
Medication	Yes, Previously/Currently = 325 (27.7%)	13.18 ± 6.53	11.19 ± 5.88	3.06 ± 0.28	9.39 ± 3.15	62.49 ± 23.13	40.76 ±32.37
	No = 848 (72.3%)	10.04 ± 5.80	7.98 ± 5.52	3.05 ± 0.32	7.34 ± 2.35	72.20 ± 18.91	25.76 ±28.09
	T-test significance	***	***	ns	***	***	***
Healthcare service	Yes = 401 (34.2%)	12.38 ± 6.50	10.42 ± 5.99	3.03 ± 0.30	8.86 ± 3.20	64.25 ± 22.33	40.03 ±32.07
	No = 772 (65.8%)	10.14 ± 5.86	8.06 ± 5.53	3.07 ± 0.32	7.42 ± 2.36	72.24 ± 19.13	24.66 ±27.59
	T-test significance	***	***	*	***	***	***

**Table 9:** Bivariate analysis on COVID-related questions.

Variables	Categories	PHQ9 Depression	GAD7 Anxiety	BRS6 Resilisence	EQ5D5L Life quality	Health score	Support urgency
IV. COVID-related questions							
Social distancing	Never/Rarely/Sometimes = 77 (6.6%)	11.95 ± 6.49	8.74± 5.39	3.06 ± 0.32	8.10 ± 3.09	65.61 ± 25.79	33.71 ± 33.10
	Very often/Always = 1096 (93.4%)	10.83 ± 6.15	8.88± 5.83	3.06 ± 0.31	7.89 ± 2.73	69.78 ± 20.20	29.65 ± 29.86
	T-test significance	ns	ns	ns	ns	ns	ns
Risk Group	High/Increased risk = 175 (14.9%)	11.90 ± 6.43	9.26 ± 5.85	3.03 ± 0.30	7.67 ± 2.45	63.43 ± 23.97	28.17 ± 29.94
	None = 998 (85.1%)	10.73 ± 6.12	8.80 ± 5.79	3.06 ± 0.30	9.24 ± 3.83	70.58 ± 19.80	39.90 ± 34.32
	T-test significance	*	ns	ns	***	***	***
Living Group	Yes = 353 (30.1%)	11.90 ± 6.43	9.63 ±5.78	3.06 ± 0.33	8.26 ± 2.71	66.99 ± 20.33	33.69 ±30.65
	No = 820 (69.9%)	10.48 ± 6.01	8.54 ± 5.78	3.05 ±0.31	7.75 ± 2.76	70.59 ± 20.66	28.29 ± 29.70
	T-test significance	***	*	ns	**	**	**
Contract risk	Extremely unlikely(1)/unlikely(2)/Neutral(3) = 979 (83.5%)	10.76 ± 6.20	8.69 ± 5.82	3.06 ± 0.31	7.85 ± 2.74	70.44 ± 20.13	28.02 ± 29.40
	Likely(4)/Extremely likely(5) = 194 (16.5%)	11.64 ± 6.02	9.47 ± 5.64	3.02 ±0.32	8.19 ± 2.84	64.82 ± 22.42	39.50 ± 31.71
	T-test significance	ns	*	ns	ns	***	***

## Results

A total of 1173 valid responses were collected without any missing values (each of the 48 asked questions was required to fill in before the survey could be submitted).

### Descriptive analysis

Table 1 presents the descriptive analysis for demographic related variables. The mean of age was 25.65 (standard deviation = 8.89), with a median value of 22 and 70% of participates were females. Approximately one third of the students were living in the University town, with 23% living in nearby cities and 43% living in various other places. Nearly one third of the students were from non-white ethnic backgrounds and there were slightly more (53%) post graduates than undergraduates. 45% were single and 54% in a relationship.

Table 2 summarises the descriptive statistics regarding the life style/living situation, including the experience of an adverse life event during the pandemic. Regarding adversity, 979 out of the 1173 (86.6%) participants reported at least one adverse effect of the pandemic, with an average of 2.3 adverse effects per student. The three most prevalent adverse effects were the cancellation of an important event (56.1%), worse personal relations (40.2%) and worse financial situation (39.6%). Over 13% of students also reported the death of a partner, close relative or friend. Approximately 30% of students were exercising frequently despite lockdown, 18% reported often or always using alcohol and 12% often or always using tobacco. 72% reported that their relationships with friends or families had been impacted and 60% had often or always been communicating with friends and family remotely.

Table 3 shows high levels of use of mental health and talking therapies, with approximately 40% of students having been referred to or participated in talking therapies for a mental health issue in the past (not just at the current time), 27.7% reported currently or previously taking medications for a mental health issue and about a third had accessed or attempted to access healthcare services personally or for a family member during the pandemic period. Table 4 shows that over 90% of the students reported practicing the recommended social distancing often/always, 15% believed they were in high or increased risk groups for pre-existing health conditions or needing special medical care, with 30% living with another person in the high risk groups, while 16.5% of students felt they were likely or extremely likely to contract COVID-19 virus.

Table 5 shows the descriptive analysis on decision variables. The mean value of PHQ9 (depression) was in the moderate range and the mean GAD7 score (anxiety) was in the mild range; the BRS6 measure of resilience was very slightly above the lower boundary of the range [3.00 to 4.30], just within the average level of normal resilience. The EQ-5D-5L mean of 7.9 is relatively close to the best possible value of 5, suggesting good quality of life for the sample as a whole. The mean self-rated health score, which is part of the EQ-5D-5L instrument, was 69.51, but with a large standard deviation suggesting a wide variation across the sample. The perceived support needs value of 29.9 suggests relatively low levels of urgent support was required during lockdown across the sample.

Table 10 shows distribution of PHQ9, GAD7 and BRS6 scores across the severity levels and table VII shows the numbers and percentages of students who scored above and below the clinical cut-offs on the PHQ9 and GAD7, as a whole sample and by gender. Table 11 shows 53.4% of students scored in the clinical range for depression on the PHQ9 (suggestive of clinically significant depression), with females being more likely (56.8%) than males (43.3%) to be above the cut-off. 51.5% of the students scored above the anxiety cut off (suggestive of clinically significant anxiety) on the GAD7 and again the percentage was higher for females (54.8%) than for males (43.2%). The BRS6 scores suggest 26% were in the low resilience category, with no significant gender difference. No students were in the high resilience category.

**Table 10:** Detailed distributions of PHQ9, GAD7 and BRS6.

PHQ9	**[0-4](none)**		**[5-9](mild)**		**[10-14](moderate)**		**[15-19](moderately severe)**		**[20-27](severe)**	
	#	%	#	%	#	%	#	%	#	%
Male	85	25.0%	101	29.7%	72	21.2%	54	13.1%	28	8.2%
Female	99	12.0%	258	31.2%	220	26.6%	166	20.1%	83	10.0%
Whole	184	15.8%	359	30.8%	292	25.0%	220	18.9%	111	9.5%
GAD7	**[0-5](none)**		**[6-10](mild)**		**[11-15](moderate)**		**[16-21](severe)**			
	#	%	#	%	#	%	#	%		
Male	144	42.4%	97	28.5%	51	15.0%	48	14.1%		
Female	236	28.6%	272	32.9%	157	19.0%	161	19.5%		
Whole	380	32.6%	369	31.6%	208	17.8%	209	17.9%		
BRS6	**[1, 2.99](low resilience)**		**[3.00, 4.30] (normal resilience)**		**[4.31, 5.00](high resilience)**					
	#	%	#	%	#	%				
Male	92	27.1%	248	72.9%	0	0%				
Female	215	26.0%	611	74.0%	0	0%				
Whole	307	26.3%	859	73.7%	0	0%				

**Table 11:** Distributions of PHQ9 and GAD7 based on clinical thresholds and gender.

PHQ9	[<10]		[>= 10]	
	#	%	#	%
Male	186	54.7%	154	45.3%
Female	357	43.2%	469	56.8%
Whole	543	46.6%	623	53.4%
GAD7	[<8]		[>= 8]	
	#	%	#	%
Male	193	54.7%	147	43.2%
Female	373	45.2%	453	54.8%
Whole	566	48.5%	600	51.5%

### Bivariate associations analysis with decision variables

Bivariate associations between demographic characteristics and mental health variables are presented in Table 6. With respect to gender, female students had significantly higher levels of anxiety and depression and poorer overall self-rated health. Comparing scores for undergraduates and post-graduates, a statistically significant difference was found for the PHQ-9 (depression) only, with undergraduates showing a higher level of depression. This is also reflected in a higher percentage of undergraduates scoring above the clinical cut off on the PHQ9 (56.4%) compared to post-graduates (51%), shown in Table 12. No significant differences were found in relation to any decision variable when comparing those who were single or in a relationship.

**Table 12:** Distributions of PHQ9 and GAD7 based on clinical thresholds and education level.

PHQ9	[<10]		[> = 10]	
	#	%	#	%
Undergrads	240	43.6%	311	56.4%,
Postgrads	305	49.0%	317	51.0%
Whole	545	46.5%	628	53.5%
GAD7	[<8]		[> = 8]	
	#	%	#	%
Undergrads	263	47.7%	288	52.3%
Postgrads	306	49.2%	316	50.8%
Whole	569	48.5%	604	51.5%

Bivariate associations between lifestyle/living situations and mental health burden variables are presented in Table 7. Regarding adverse life experiences during the pandemic, there were no statistically significant differences in depression, anxiety or quality of life between those reporting a negative impact of the pandemic on their life compared to those without any. Interestingly, students reporting at least one impact showed higher level of resilience and self-rated health scores but lower support needs. Regarding exercising, those who always or very often exercised showed lower levels of depression and anxiety, better quality of life, higher self-rated health score and lower support needs, compared to those who occasionally or never exercised.

Regarding the consumption of alcohol and tobacco, most students reported using either substance less often during the pandemic. Alcohol use was not related to any of the decision variables but tobacco use was significantly related to five decision variables, with those using tobacco showing higher anxiety and depression, lower life quality of life and self-reported health, and higher support needs.

Those students reporting an impact of the pandemic on their relationships tended to report higher scores of depression and anxiety, significant enough to put the affected cohort in one level up in terms of severity for both depression and anxiety. This also applied to students reporting scarce communication with friends and family. Those whose relationships had not been impacted or who had maintained good communication with family and friends tended to have better quality of life and self-reported health and lower support needs.

The bivariate analysis of the students’ mental wellbeing history against the set of decision variables are presented in Table 8. Students who had been referred to or participated in talking therapies for a mental health issue in the past (not just at the current time), tended to report higher scores of depression and anxiety, significant enough to put the affected cohort in one level up in terms of severity for both depression and anxiety. Students who reported currently or previously taking medications for a mental health issue, or had accessed or attempted to access healthcare services personally or for a family member during the pandemic period, also tended to experience higher levels of depression and anxiety with lower life quality and self-rated health score and more support needs.

The bivariate analysis on COVID-19 related questions against the set of decision variables are presented in Table 9. A large majority of participants, 93.4%, reported always or very often practising social distancing and this factor was not related to any of the decision variables (possibly due to the highly skewed distribution on this factor). About 15% of the students were in high or increased risk due to pre-existing medical conditions or needing special care and the higher risk group showed higher scores of depression and lower health scores, yet they had lower support needs and better index for quality of life. Furthermore, approximately 30% of the students reported living with someone in a high or increased risk group and these tended to showed higher levels of depression and anxiety, poorer quality of life and lower self-rated health scores, while having more support needs. Finally, 16.5% of the participants reported feeling likely or extremely likely to contract the virus, and these showed an increased level of anxiety, worse health score and higher support needs.

### Multivariate linear regression analysis

A multivariate linear regression was conducted for each of the dependent variables, with results presented in Tables 13 and 14, with each regression analysis using a different subset of independent variables, those which were found to be statistically significantly related from the bivariate analysis as shown in Tables 6–9.

**Table 13:** Regression analysis, where *ns* = non- significant(*p* > 0.05), *(*p* ≤ 0.05);**(*p* ≤ 0.01);***(*p* ≤ 0.001).

	PHQ9 Depression		GAD7 Anxiety		BRS6 Resilisence	
Variables	*β*	95%CI	*β*	95%CI	*β*	95%CI
I. Demographics						
Gender	-0.0669	[-0.119, -0.015]**	-0.0844	[-0.137, -0.032]**	-	-
Ethnicity	-	-	-	-	-0.0773	[-0.134, -0.020]**
Education	-0.0610	[-0.113, -0.009]*	-	-	-	-
Relationship	-	-	-	-	-	-
II. Lifestyle / living situation						
Adversity	-	-	-	-	0.0590	[0.002, 0.116]*
Exercise	-0.1802	[-0.232, -0.128]***	-0.0996	[-0.152, -0.048]***	-	-
Alcohol	-	-	-	-	-	-
Tobacco	0.1041	[0.052, 0.156]***	0.1110	[0.059, 0.163]***	-	-
Relationship impact	0.1338	[0.082, 0.186]***	0.1300	[0.078, 0.182]***	-	-
Communication	-0.1732	[-0.225, -0.121]***	-0.1789	[-0.231, -0.126]***	-	-
III. Mental wellbeing history						
Talking therapy	0.1360	[0.071, 0.201]***	0.1701	[0.105, 0.235]***	-	-
Medication	0.0753	[0.011, 0.140]*	0.0729	[0.008, 0.138]*	-	-
Healthcare service	0.1037	[0.050, 0.157]***	0.1202	[0.066, 0.174]***	-0.0535	[-0.111, 0.004]ns
IV. COVID-related questions						
Social distancing	-	-	-	-	-	-
Risk Group	0.0217	[-0.030, 0.074]ns	-	-	-	-
Living Group	0.0736	[0.021, 0.126]**	0.0625	[0.011, 0.114]*	-	-
Contact Risk	-	-	0.0291	[-0.023, 0.081]ns	-	-
Test Statistics	*R*^2^ = 0.208;	F- statistic = 27.69; *p* < 0.001	*R*^2^ = 0.202;	F- statistic = 29.37; *p* < 0.001	*R*^2^ = 0.014;	F- statistic = 5.346; *p* < 0.01

**Table 14:** Regression analysis (cont’d), where *ns* = non- significant(*p* > 0.05), *(*p* ≤ 0.05);**(*p* ≤ 0.01);***(*p* ≤ 0.001).

	EQ5D5L Life quality		Health score		Support needs	
Variables	*β*	95%CI	*β*	95%CI	*β*	95%CI
I. Demographics						
Gender	-	-	0.0321	[-0.022, 0.086]ns	-	-
Ethnicity	-	-	-	-	-0.0946	[-0.150, -0.039]***
Education	-	-	-	-	-	-
Relationship	-	-	-	-	-	-
II. Lifestyle / living situation						
Adversity	-	-	0.0325	[-0.021, 0.086]ns	-0.0227	[-0.077, 0.031]ns
Exercise	-0.1414	[-0.191,-0.091]***	0.1752	[0.122, 0.228]***	-0.0794	[-0.133, -0.026]**
Alcohol	-	-	-	-	-	-
Tobacco	0.0689	[0.019, 0.119]**	-0.0824	[-0.136, -0.029]**	0.0435	[-0.010, 0.097]ns
Relationship impact	0.1092	[0.059, 0.159]***	-0.0393	[-0.093, 0.014]ns	0.0040	[-0.050,0.058]ns
Communication	-0.1284	[-0.179, -0.078]***	0.1379	[0.084, 0.191]***	-0.1047	[-0.159, -0.050]***
III. Mental wellbeing history						
Talking therapy	0.1906	[0.128, 0.253]***	-0.1557	[-0.222, -0.090]***	0.0830	[0.016, 0.150]*
Medication	0.1445	[0.082, 0.207]***	-0.0466	[-0.113, 0.020]ns	0.1248	[0.057, 0.192]***
Healthcare service	0.1534	[0.102, 0.205]***	-0.1127	[-0.168, -0.058]***	0.1760	[0.121, 0.231]***
IV. COVID-related questions						
Social distancing	-	-	-	-	-	-
Risk Group	0.1474	[0.097, 0.198]***	-0.0771	[-0.130, -0.024]**	0.0786	[0.025, 0.133]**
Living Group	0.0437	[-0.006, 0.094]ns	-0.0503	[-0.103, 0.003]ns	0.0433	[-0.010, 0.097]ns
Contact Risk	-	-	-0.0555	[-0.109, -0.002]*	0.1024	[0.048, 0.156]***
Test Statistics	*R*^2^ = 0.259;	F- statistic = 45.30; *p* < 0.001	*R*^2^ = 0.168;	F- statistic = 19.56; *p* < 0.001	*R*^2^ = 0.151;	F- statistic = 17.23; *p* < 0.001

Apart from the regression model for the BRS6 Resilience variable, which achieves the statistical significance at the level of *p* < 0.01 (with *R*^2^ only being 0.014), the remaining regression models all achieve statistical significance with *p* < 0.001, indicating the selection of underlying variables are meaningful in associating with the level of the corresponding decision variable.

Ten out of eleven variables included for the analysis of the PHQ9 show statistical significance. Exercise was the predictor with biggest coefficient, closely followed by communication, and then the history of accessing a talking therapy and impact on relationships. The values of these coefficients within 95% of the data, (the confidence interval, CI), or within two standard deviations, are also listed accordingly. For the regression analysis for the GAD7, ten variables were included with only the contract risk not showing a statistical significance contribution. The level of communication with families/friends was the most significant predictor for anxiety, followed by accessing a talking therapy and impact on relationships. There were also significant gender differences, with female students showing higher levels of anxiety and depression, and lower self-rated health scores. For BRS6, only three independent variables were included, with ethnicity and the adversity question (which asked whether the student had experienced any adverse events) showing statistical significance.

Nine predictors were included for the analysis of EQ5D5L quality of life measure, with the three most significant predictors being a history of talking therapy, taking medication for a mental health issue and access or attempt to access healthcare service personally or for a family member during the pandemic.

Twelve predictors were included for the analysis of self-rated health score, with seven showing a statistically significant correlation. Exercise, accessing talking therapy and communication were the top three predictors.

Eight out of twelve variables were significant predictors for the support needs dependent variable, with healthcare service, medication and communication being top three predictors.

## Discussion

This survey set out to investigate the mental health and wellbeing of higher education students during the COVID19 pandemic and predictive factors. Perhaps the most striking finding was the high levels of depression and anxiety, with scores above the clinical cut off for over half the students. This suggests they are likely to have been experiencing clinically significant levels of depression and/or anxiety at the time of the survey. Measures such as the PHQ9 and GAD7 are screening instruments which highlight individuals likely to have significant problems and requiring further assessment and support. Although these measures are not diagnostic, they have been favourably compared to diagnostic interviews [22]. Previous community surveys suggest about one in six people report experiencing a common mental health problem (anxiety or depression) in a given week in England [2] so our survey suggests the incidence of common mental health problems was much higher than usual in this student group during the pandemic. The survey took place over one month, about three to four months after the first lockdown in the UK began (and after exam time for undergraduates), so it is likely to be due to the pandemic rather than other factors but we do not know how the levels of anxiety and depression changed over time. A recent study [23] identified different trajectories of depression and anxiety symptoms over time for subgroups in a large community sample in England during the pandemic. For example, they found young, female and more sociable people, and essential workers, experienced severe anxiety at the start of the lockdown which quickly decreased whilst younger people with lower incomes and previous mental health problems experienced increasing levels of symptoms over time. Other surveys have also showed increases in common mental health problems during the pandemic. For example, an Office for National Statistics (ONS) survey in early 2021 reported about 1 in 5 (21%) adults experienced depression in early 2021, more than double that found before the COVID-19 pandemic (10%). This ONS survey also reported that younger adults and women were more likely to experience some form of depression, with 43% of women and 26% of men in the 16 to 29 year age range found to be experiencing depressive symptoms. This higher level of depressive symptoms in women and younger adults is consistent with our survey and we also found higher levels of anxiety symptoms. Other studies have also reported higher levels psychological distress in younger people, with higher levels in females compared to males, during the pandemic [24]. The high percentages of students reporting current or past referral to or receiving talking therapy (40%) and/or medication for mental health problems (28%) suggest this may have been a sample with relatively high levels of pre-existing mental health difficulties but this is speculative. The high levels of anxiety and depression contrast to some extent with the quality of life scores, which might suggest that the experience of low mood and anxiety were due to the current circumstances of the pandemic (and therefore transient for many people), rather than longer term mental health difficulties that had impacted on quality of life. However, we do not how, as the pandemic and restrictions persisted, quality of life was affected in the longer term. It was interesting that the 15% of the students who were in high or increased risk due to pre-existing medical conditions or needing special care showed higher scores of depression and lower health scores, yet they had lower support needs and better index for quality of life. It is possible that this was due to them having better support systems in place before the pandemic, which they continued to access.

The study also set out to investigate levels and predictors of resilience and most students scored in the normal range with 26% in low resilience category and none in the high category. This is reflected in the overall mean of 3.1 which is lower than that reported in the original validation study [19] which reported on four samples (including two student samples), with means ranging from 3.53 to 3.98 and lower scores in the younger age groups. An interpretation of the resilience scores is that the pandemic not only led to increases in anxiety and depression but also undermined personal resilience. For example, the restrictions of lockdown were likely to have deprived students of outlets and activities important to their wellbeing. Also, low mood may have contributed to a negative appraisal of personal resilience at the time. It is therefore possible that the low resilience is due to the adverse circumstances of the pandemic and lockdown, not stable characteristics of the individuals. We suggest our findings should be interpreted in terms of attributing the distress and coping as consequence of the situation the students found themselves in, ‘what happened to them’ during the pandemic, rather than ‘what is wrong with’ them [25, 26].

The survey also suggested a number of factors that may have contributed to the high levels of common mental health problems (in addition to gender), such as cancellation of an important event (56%), worse personal relations (40%) and worse financial situation (40%). Students with higher levels of depression tended to exercise less, communicated less with friends/family, and experienced greater impacts on their relationships, as well as (not surprisingly) having more history of accessing a talking therapy. Those with higher anxiety levels also tended to communicate less with friends/family, experience greater impacts on their relationships, and had more history of accessing a talking therapy. We cannot attribute causal effects and in some cases the relationships between these variables are likely to work both ways. For example, low mood may lead to worse personal relationships which would adversely affect mood.

The relationship between exercise and mental health in our survey is consistent with evidence that physical activity has a role in preventing depression [27] and a physical activity programme is recommended for people with mild to moderate depression [28]. The finding that tobacco use was significantly related to higher anxiety and depression, lower life quality of life and self-reported health, and higher support needs is consistent with reports that as the severity of mental health problems increase, the prevalence of smoking is higher [29]. For example, Public Health England reports that whilst the prevalence of smoking for all adults in England was 16.4% in 2014 to 2015, the prevalence was 28% for people with anxiety or depression, and 40.5% for those with serious mental illness. There is also good evidence that smoking cessation is associated with reductions in anxiety and depression, and with improvements in mood and quality of life, and that this applies to those with mental health problems [30].

There are a number of limitations of this study. Firstly, this was a one off, cross sectional survey over one month, four months into the pandemic so we are not able to report on changes over time, and crucially, whether the high levels of anxiety and depression were transitory or longer term. Secondly, the survey was carried out at only one University in England so it is possible findings will differ in other localities. Having said that, there was diversity in our sample, which included students from all over the UK with a higher proportion of non-white students (32%) than in the general population in England, enabling comparisons to be made between white and non-white students. Thirdly, it is possible that there was a bias in our sample, in that those experiencing mental health difficulties at the time of the survey were more likely to be attracted by the advert for the survey and to complete it. It may not therefore be representative of the larger population of students but the high levels of anxiety and depression and gender difference are nevertheless significant findings. Finally, although the study identifies levels and predictors of mental health distress, it does not identify how the pandemic impacted on individuals, and how they coped with the challenges. This would require more in depth, qualitative research and we recommend this to complement surveys.

Despite these limitations, this study identifies high levels of anxiety and depression in undergraduate and postgraduate students early in Covid-19 pandemic in England and relatively low levels of resilience which are likely to reflect the impact of restrictions and isolation which reduced the opportunities to engage in helpful coping strategies and activities. These high levels of psycho logical distress are therefore likely to be a combination of pandemic-related stresses and limited access to positive coping behaviours, such as socialising. Mental health distress was associated with lower levels of exercising, higher levels of tobacco use, and a number of life events likely to be associated with the pandemic and lockdown, such as cancelled events, worsening in personal relationships and financial concerns. We can assume that some of the students would have been experiencing high levels of depression and anxiety for the first time, whilst others would have experienced a worsening of pre-existing difficulties. This study highlights how these mainly younger adult students may be particularly prone to experiencing high levels of anxiety and low mood during a pandemic, and the importance of providing support to reduce the likelihood of longer-term problems. Given the global context for these increased levels of psychological distress, it is important to see this as an understandable reaction to an adverse situation which may be transitory, rather than necessarily the start of continued and long-term problems but this will only be achieved with support. Some will find adequate support within their social networks, coping strategies and activities, but it is likely a significant number would be at risk of longer term and more severe difficulties and might therefore benefit from professional support including psychological therapy and counselling.
